# Volatile Composition, Antimicrobial Activity, and In Vitro Innate Immunomodulatory Activity of *Echinacea purpurea* (L.) Moench Essential Oils

**DOI:** 10.3390/molecules28217330

**Published:** 2023-10-29

**Authors:** Noura S. Dosoky, Liliya N. Kirpotina, Igor A. Schepetkin, Andrei I. Khlebnikov, Brent L. Lisonbee, Jeffrey L. Black, Hillary Woolf, Trever L. Thurgood, Brittany L. Graf, Prabodh Satyal, Mark T. Quinn

**Affiliations:** 1 Essential Oil Science, dōTERRA International, 1248 W 700 S, Pleasant Grove, UT 84062, USA; ndosoky@doterra.com; 2 Department of Microbiology and Cell Biology, Montana State University, Bozeman, MT 59717, USA; kirpotina@hotmail.com (L.N.K.); igor@montana.edu (I.A.S.); 3 Kizhner Research Center, Tomsk Polytechnic University, Tomsk 634050, Russia; aikhl@chem.org.ru; 4 Innova Bio, Utah Valley University, 800 W University Pkwy, Orem, UT 84058, USA; brentleelisonbee@gmail.com (B.L.L.); black.jeffrey21@gmail.com (J.L.B.); trever.l.thurgood@gmail.com (T.L.T.); 5 Research and Development, dōTERRA International, 389 S 1300 W, Pleasant Grove, UT 84062, USA; hwoolf@doterra.com (H.W.); blgraf@doterra.com (B.L.G.)

**Keywords:** *Echinacea purpurea*, purple coneflower, essential oil, antimicrobial activity, (+)-δ-cadinene, calcium flux, neutrophil, chemotaxis

## Abstract

*Echinacea purpurea* (L.) Moench is a medicinal plant commonly used for the treatment of upper respiratory tract infections, the common cold, sore throat, migraine, colic, stomach cramps, and toothaches and the promotion of wound healing. Based on the known pharmacological properties of essential oils (EOs), we hypothesized that *E. purpurea* EOs may contribute to these medicinal properties. In this work, EOs from the flowers of *E. purpurea* were steam-distilled and analyzed by gas chromatography–mass spectrometry (GC–MS), GC with flame-ionization detection (GC–FID), and chiral GC–MS. The EOs were also evaluated for in vitro antimicrobial and innate immunomodulatory activity. About 87 compounds were identified in five samples of the steam-distilled *E. purpurea* EO. The major components of the *E. purpurea* EO were germacrene D (42.0 ± 4.61%), α-phellandrene (10.09 ± 1.59%), β-caryophyllene (5.75 ± 1.72%), γ-curcumene (5.03 ± 1.96%), α-pinene (4.44 ± 1.78%), δ-cadinene (3.31 ± 0.61%), and β-pinene (2.43 ± 0.98%). Eleven chiral compounds were identified in the *E. purpurea* EO, including α-pinene, sabinene, β-pinene, α-phellandrene, limonene, β-phellandrene, α-copaene, β-elemene, β-caryophyllene, germacrene D, and δ-cadinene. Analysis of *E. purpurea* EO antimicrobial activity showed that they inhibited the growth of several bacterial species, although the EO did not seem to be effective for *Staphylococcus aureus*. The *E. purpurea* EO and its major components induced intracellular calcium mobilization in human neutrophils. Additionally, pretreatment of human neutrophils with the *E. purpurea* EO or (+)-δ-cadinene suppressed agonist-induced neutrophil calcium mobilization and chemotaxis. Moreover, pharmacophore mapping studies predicted two potential MAPK targets for (+)-δ-cadinene. Our results are consistent with previous reports on the innate immunomodulatory activities of β-caryophyllene, α-phellandrene, and germacrene D. Thus, this study identified δ-cadinene as a novel neutrophil agonist and suggests that δ-cadinene may contribute to the reported immunomodulatory activity of *E. purpurea*.

## 1. Introduction

The genus *Echinacea* L. (Asteraceae) contains ten species generally known as coneflowers. Coneflowers have big, showy heads of composite flowers with spiny central disks that bloom throughout the summer in Europe and North America. *Echinacea purpurea* (L.) Moench (purple coneflower) is a well-known medicinal-ornamental perennial plant native to damp prairies, meadows, and open forests of the central to southeastern parts of the United States (Ohio, Michigan, Iowa, Louisiana, and Georgia) [1]. Due to its pharmacological importance, *Echinacea* is cultivated worldwide. It typically grows from 2 to 4’ tall and has coarse, ovate to broad-lanceolate, dark green leaves. *Brauneria purpurea* (L.) Britton, *Echinacea intermedia* Lindl. Ex Paxton, *Echinacea serotina* (Sweet) D. Don ex G. Don f., *Echinacea speciosa* (Wender.) Paxton, *Helichroa purpurea* (L.) Raf., and *Rudbeckia purpurea* L. are known botanical synonyms of *E. purpurea* [2].

*E. purpurea* preparations are among the best-selling herbal supplements [3]. Historically, *E. purpurea* has been used in the treatment of upper respiratory tract infections [4], common colds [5], sore throats, migraines, colic, stomach cramps, and toothaches [6] and to promote wound healing [7]. Likewise, extracts of various parts of *E. purpurea* have been reported to exhibit antioxidant, anti-inflammatory, anxiolytic, immunoregulatory, antiproliferative, antiviral, antibacterial, and antifungal properties [1,8,9,10,11,12,13,14]. This plant has a reputation for stimulating the immune system to help fight infectious diseases. Standardized preparations of *E. purpurea* have several applications for respiratory tract infections [5,15,16,17,18,19,20,21,22,23], viral infections [24,25,26,27], tumor suppression [28], acne, and skin diseases [29,30]. In addition, a mixture of *E. purpurea* and *E. angustifolia* was reported to protect against acetic acid-induced ulcerative colitis in rats [31]. Attempts to use *Echinacea* as an immune modulator date back over a century, with early experiments revealing its potential to stimulate the immune system. *Echinacea*’s immunological properties have been extensively studied since 1923 [32]. Early studies confirmed phagocyte-stimulating, hyaluronidase-inhibiting, and properdin-generating activities. Experiments using all three medicinal species of *Echinacea* have demonstrated macrophage-activating properties [33], including reports of increased macrophage phagocytic activity accompanied with enhanced cytokine production, in vitro and in vivo [33]. *E. purpurea* EOs have been shown to exhibit anti-inflammatory effects in mice and rats [12,34], and EOs from various medicinal plants have been reported to exhibit immunomodulatory activity through their ability to modulate neutrophil functional activity. Since neutrophils perform essential host defense functions, they represent an ideal pharmacological target for therapeutic development [35]. Likewise, the antimicrobial properties of EOs have been reported in several studies (reviewed in [36]). Thus, we propose that a combination of antimicrobial activity with innate immunomodulatory activity may represent an ideal approach for protection against pathogens while modulating the ensuing inflammatory response and suggest that such EOs may offer promise as an alternative treatment option.

The efficacy of *E. purpurea* preparations has been investigated in several clinical trials, but the results varied greatly depending on the plant parts used, the extract type, the variability of active components, and the sample size. Previous studies identified several bioactive compounds from ethanolic and water extracts of *E. purpurea*, including caffeic acid derivatives (caffeic acid, caftaric acid, cichoric acid, echinacoside, chlorogenic acid, and cynarin), alkylamides, flavonoids (rutin, quercetin, nicotiflorin, and luteolin), polysaccharides, and polyacetylenes [37,38,39,40,41,42,43]. These compounds have been extensively studied and are thought to be responsible for the bioactivity of *E. purpurea* extracts. Although a few studies investigated the *E. purpurea* EO, little is known about their biological activities. Based on previous studies demonstrating immunomodulatory activity of EOs, we hypothesized that the *E. purpurea* EO may contribute to the medicinal properties of extracts from this plant. Thus, the current study aims to explore the volatile composition of EOs extracted from the flowers of *E. purpurea* from Bulgaria and evaluate their in vitro antibacterial activity and innate immunomodulatory potential in human neutrophils.

## 2. Results and Discussion

### 2.1. Chemical Composition

**Figure 1:**
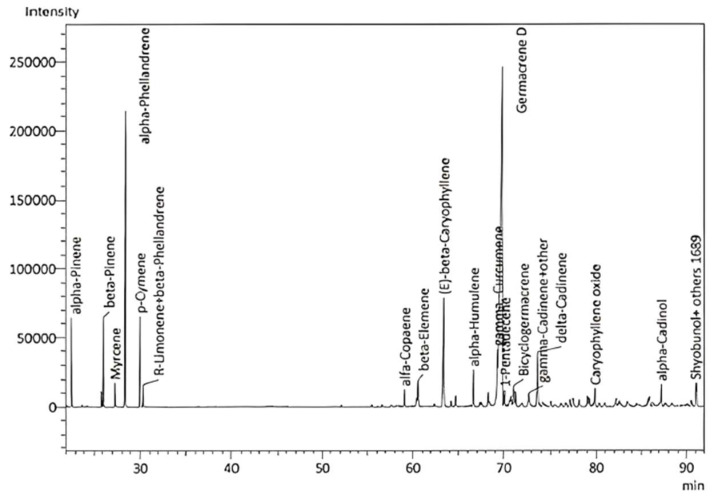
GC–MS chromatogram of *E. purpurea* flower EO.

Five *E. purpurea* samples (E1–5) were steam-distilled for 3–4 h in a Clevenger-type apparatus. The average yield was 0.13 ± 0.06%. The EO had a deeply sweet, herbaceous, floral, slightly grassy, and lightly hay-like scent and was analyzed by GC–MS and GC–FID to identify and quantify the EO component compounds, respectively (Figure 1). A total of 87 compounds were identified, representing 97.65–100% of the total EO composition (Table 1). Note, it has been reported that the volatile components can be detected in the aerial parts and roots of *E. purpurea*, with variable yields and chemical compositions that can be affected by pathogen attacks [44]. The major components of the *E. purpurea* EO in this study were germacrene D (42.0 ± 4.61%), α-phellandrene (10.09 ± 1.59%), β-caryophyllene (5.75 ± 1.72%), γ-curcumene (5.03 ± 1.96%), α-pinene (4.44 ± 1.78%), δ-cadinene (3.31 ± 0.61%), and β-pinene (2.43 ± 0.98%) (values indicate % of the EO). The abundance of germacrene D correlates well with previous reports on essential oils from the flowerheads of *E. purpurea* [45,46,47]. Interestingly, analysis of the flowerheads revealed that α- and β-pinene, β-myrcene, ocimene, limonene, camphene, and terpinene were the main components [48]. The hydrodistilled EO of cultivated *E. purpurea* flowerheads during ontogenesis comprised mainly β-caryophyllene (25.0%), fatty acids (17.2%), germacrene D (13.8%), α,β-pinene (9.5%), nerolidol (6.6%), and α-phellandrene (4.2%) [49].

### 2.2. Enantiomeric Distribution 

The enantiomeric distribution of chiral compounds in *E. purpurea* EO is presented in Table 2. The results revealed a total of 11 chiral compounds: α-pinene, sabinene, β-pinene, α-phellandrene, limonene, β-phellandrene, α-copaene, β-elemene, β-caryophyllene, germacrene D, and δ-cadinene. (−)-α-phellandrene, (+)-α-copaene, and (−)-β-caryophyllene appeared as pure enantiomers. Thus, these chiral constituents and their enantiomeric distributions could be used as reference standards for identifying adulteration in *E. purpurea* EO.

### 2.3. Antimicrobial Activity of E. purpurea EO 

It has been previously published that extracts from *E. purpurea* had MIC values of 93.8 mg/mL against *S. mutans* and 375 mg/mL against *E. coli* [50]. It is likely that much of the antimicrobial activity of the *E. purpurea* EO is derived from the germacrene D content, as the *Echinacea* EO has been shown to contain about 42% germacrene D [51]. As shown in Table 3, the *E. purpurea* EO inhibited the growth of several bacterial species, although the EO did not seem to be effective against *S. aureus* and had lower activity for *E. coli* and *S. epidermidis.* The major constituents contributing to the antimicrobial activity of the *E. purpurea* EO are likely β-caryophyllene and α-pinene, which have both exhibited potent antimicrobial activities as single compounds in vitro [36,52,53,54,55,56]. The other major constituents in the *E. purpurea* EO, including germacrene D, α-phellandrene, β-pinene, γ-curcumene, and δ-cadinene, may provide additive or synergistic antimicrobial effects, as these molecules, or EOs derived from other plants that contain high levels of these molecules, have been reported to exhibit moderate antimicrobial activities [56,57,58,59,60,61,62,63,64].

### 2.4. Innate Immunomodulatory Activity of E. purpurea EO and Its Components 

**Figure 2:**
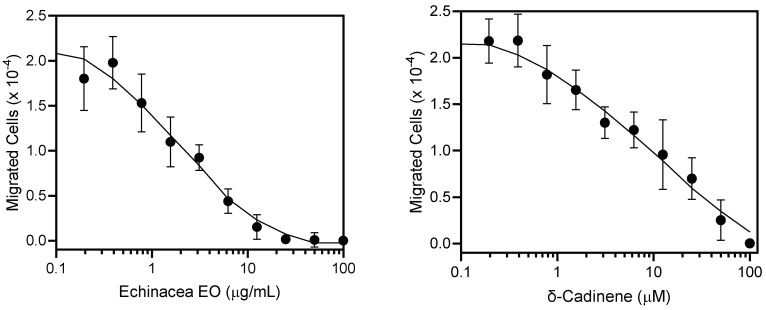
Inhibition of neutrophil chemotaxis by *Echinacea purpurea* EO and (+)-δ-cadinene. Neutrophil chemotaxis toward 1 nM *f*MLF was measured, as described under Section 3. The data are presented as the mean ± SEM and are based on two independent experiments.

A growing body of research suggests that *E. purpurea* has immunostimulatory properties. For example, the *E. purpurea* root extract was reported to have immune-enhancing properties by lowering the frequency and function of regulatory T cells [65]. In another study, oral administration of an *E. purpurea* extract increased natural killer cell activity in mice by increasing the levels of MHC II, CD4 T cells, and Th1 cytokines [66]. Likewise, an ethanolic extract of the aerial parts was reported to modulate cytokine response in human T-cells [67]. We hypothesized that the *E. purpurea* EO could have innate immunomodulatory properties. In the present study, human neutrophils were used to evaluate the innate immunomodulatory effects of the *E. purpurea* EO and their major components. Neutrophils are immune cells that serve as both effectors and regulators in the development of the innate inflammatory response [68]. As shown in Table 4, the treatment of human neutrophils with the *E. purpurea* EO induced intracellular Ca^2+^ mobilization (EC_50_ = 19.9 ± 4.5 μg/mL). This is an important finding, as intracellular Ca^2+^ mobilization plays an important role in neutrophil activation and function [69,70]. As shown in Table 4, the *E. purpurea* EO components (+)-δ-cadinene and α-phellandrene also induced comparable changes in intracellular Ca^2+^ concentrations ([Ca^2+^]_i_) in neutrophils compared to that observed previously with germacrene D [71] with EC_50_ values between 20.8 and 24.6 μM. In contrast, no activity was observed for β-caryophyllene. Previously, we found that EOs and their components could inhibit neutrophil migration [71,72,73]. Thus, we analyzed the effects of the *E. purpurea* EO and (+)-δ-cadinene on neutrophil chemotaxis and found that pretreatment with the *E. purpurea* EO (IC_50_ = 1.8 ± 0.6 μg/mL) or (+)-δ-cadinene (IC_50_ = 0.48 ± 0.1 μM) for 10 min suppressed *f*MLF-induced human neutrophil chemotaxis in a dose-dependent manner (Table 4, Figure 2).

Furthermore, we tested the cytotoxicity of the *E. purpurea* EO and its components at various concentrations in human THP-1 monocytic cells during 90 min and 24 h incubation periods to ensure that the effects on neutrophil functional activity (i.e., inhibition of Ca^2+^ mobilization and cell migration) were not significantly influenced by potential toxicity. As shown in Table 4, the *E. purpurea* EO and components had no cytotoxicity after 90 min and very little cytotoxicity after 24 h, confirming that the Ca^2+^ flux and chemotaxis assays were not influenced by possible cytotoxicity.

Although determining the mechanism is beyond the scope of this study, we sought to explore the potential protein targets of (+)-δ-cadinene. Reverse-pharmacophore mapping using the molecular structure of (+)-δ-cadinene was performed to identify its potential cellular targets. Table 5 shows the top 10 human protein targets for (+)-δ-cadinene out of 300 potential targets ranked by normalized fit scores in descending order. Interestingly, two MAPKs are indicated as possible targets among the top selected targets, namely mitogen-activated protein (MAP) kinase 1 and MAP kinase-activated protein kinase 2. Since MAP kinases play an important role in neutrophil activation and host defense mechanisms (reviewed in [74]), the possibility that (+)-δ-cadinene might interact with these kinases is very interesting and warrants future investigation of this area.

## 3. Materials and Methods

### 3.1. Materials

Dichloromethane, dimethyl sulfoxide (DMSO), *N*-formyl-Met-Leu-Phe (*f*MLF), streptomycin, and penicillin were purchased from Sigma-Aldrich (St. Louis, MO, USA). Germacrene D, (+)-δ-cadinene, α-phellandrene, and β-caryophyllene were purchased from Cayman Chemical (Ann Arbor, MI, USA), Fluo-4AM was purchased from Invitrogen (Carlsbad, CA, USA). RPMI 1640 medium was purchased from Mediatech Inc., Herndon, VA, USA. Fetal bovine serum (FBS) was purchased from ATCC (Manassas, VA, USA). Hanks’ balanced salt solution (HBSS; 0.137 M NaCl, 5.4 mM KCl, 0.25 mM Na_2_HPO_4_, 0.44 mM KH_2_PO_4_, 4.2 mM NaHCO_3_, 5.56 mM glucose, and 10 mM HEPES, pH 7.4) was purchased from Life Technologies (Grand Island, NY, USA). HBSS without Ca^2+^ and Mg^2+^ is referred to as HBSS^−^; HBSS containing 1.3 mM CaCl_2_ and 1.0 mM MgSO_4_ is referred to as HBSS^+^.

### 3.2. Essential Oil Extraction 

Cultivated *E. purpurea* was harvested from Dobrich, Bulgaria, in June–July 2020. Fresh flowers were handpicked in the early morning, transferred immediately, and steam-distilled for 3–4 h in a Clevenger-type apparatus. The plant material-to-water ratio was 1:6.

### 3.3. Gas Chromatography-Mass Spectrometry (GC–MS) Analysis 

The *E. purpurea* EOs were analyzed using a Shimadzu GC–MS-QP2010 Ultra (Shimadzu Scientific Instruments, Columbia, MD, USA) with electron impact (EI) mode with 70 eV, using 40–400 *m*/*z* range scans with a scan rate of 3.0 scan/s. The GC column used was a ZB-5ms fused silica capillary column with a (5% phenyl)-polymethylsiloxane stationary phase and a film thickness of 0.25 μm, a length of 30 m, and an internal diameter of 0.25 mm. The column temperature was set at 50 °C for 2 min and then increased by 2 °C/min to the temperature of 260 °C. The carrier gas was helium with a column head pressure of 552 kPa and a constant flow rate of 1.37 mL/min. The injector temperature was kept at 260 °C, and the ion source temperature was 200 °C. For each essential oil sample, a 1:10 *v*/*v* solution in dichloromethane (DCM) was prepared, and 0.3 μL was injected using a split ratio of 1:30. The EO components were identified by comparing mass spectral fragmentation patterns (over 80% similarity match) and retention indices (RI) based on a series of homologous C8-C20 *n*-alkanes with those reported in databases [NIST database, and our in-house library] using the Lab Solutions GC–MS post-run analysis software version 4.45 (Shimadzu Scientific Instruments, Columbia, MD, USA).

### 3.4. Gas Chromatography–Flame Ionization Detection (GC–FID) Analysis

GC–FID analysis of the *E. purpurea* EO was performed using a Shimadzu GC 2010 equipped with a flame ionization detector (Shimadzu Scientific Instruments, Columbia, MD, USA), as previously described [75], with a ZB-5 capillary column (Phenomenex, Torrance, CA, USA).

### 3.5. Enantiomeric Analysis by Chiral Gas Chromatography–Mass Spectrometry (CGC–MS)

A Shimadzu GC–MS-QP2010S with EI mode (70 eV) and B-Dex 325 chiral capillary GC column was used to perform the enantiomeric analysis of *E. purpurea* EO. Scans were in the 40–400 m/z range at a scan rate of 3.0 scan/s. The column temperature was set to 50 °C and, at first, increased by 1.5 °C/min to 120 °C and then 2 °C/min to 200 °C. The final temperature of the column was 200 °C and was kept constant. The carrier gas was helium, with a constant flow rate of 1.8 mL/min. For each EO sample, 3% *w*/*v* solution in DCM was prepared and 0.1 μL was injected using a split ratio of 1:45 [75,76]. The enantiomer percentages were determined from the peak area. A comparison of retention times and mass spectral fragmentation patterns was performed against authentic samples obtained from Sigma-Aldrich (Milwaukee, WI, USA) and was used to identify the enantiomers.

### 3.6. Isolation of Human Neutrophils

Human neutrophils were obtained using blood collected from healthy donors. Blood collection was approved by the Institutional Review Board at Montana State University (Protocol #2022-168). The isolated neutrophils were resuspended in HBSS+ for all biological assays. Neutrophil preparations were routinely >95% pure and >98% viable, as determined by light microscopy and trypan blue exclusion, respectively.

### 3.7. Cell Culture

Human THP-1 monocytic cells obtained from ATCC (Manassas, VA, USA) were cultured in RPMI 1640 medium (Mediatech Inc., Herndon, VA, USA) supplemented with 10% (*v*/*v*) FBS, 100 μg/mL streptomycin, and 100 U/mL penicillin.

### 3.8. Ca^2+^ Mobilization Assay

Changes in the intracellular Ca^2+^ concentrations ([Ca^2+^]_i_) were monitored with a FlexStation 3 (Molecular Devices, Sunnyvale, CA, USA). For these assays, neutrophils were loaded with 1.25 μg/mL Fluo-4AM and incubated in the dark at 37 °C for 30 min. The cells were then washed with HBSS^−^. The dye-loaded cells were resuspended in HBSS^+^ and pipetted into the wells of black microtiter plates at 2 × 10^5^ cells/well. To measure the direct effects of samples on [Ca^2+^]_i_, the test samples were added to the wells (final concentration of DMSO was 1%), and the fluorescence was monitored (λ_ex_ = 485 nm, λ_em_ = 538 nm). Changes in fluorescence were monitored every 5 s at room temperature for 240 s. To evaluate the inhibitory effects of the test samples, the samples were added to the wells and incubated for 10 min, with the subsequent addition of 5 nM *f*MLF. Responses were normalized to the response induced by control *f*MLF (5 nM) alone without pretreatment. These responses were assigned as 100%. To calculate median effective concentrations (EC_50_ or IC_50_), we used curve fitting (at least five or six points) and nonlinear regression analysis of the dose–response curves. Curve fitting was performed with Prism 9 (GraphPad Software, Inc., San Diego, CA, USA).

### 3.9. Chemotaxis Assay

To evaluate the effects of the *E. purpurea* EO and its components on neutrophil migration, we resuspended the neutrophils in chemotaxis media (HBSS^+^ containing 2% (*v*/*v*) heat-inactivated FBS) at 2 × 10^6^ cells/mL. We analyzed chemotaxis using 96-well ChemoTx chambers (Neuroprobe, Gaithersburg, MD). Neutrophils were first preincubated in Greiner flat-bottom 96-well plates (Millipore Sigma, Burlington, MA, USA) with different concentrations of test samples at room temperature for 30 min. To set up the chemotaxis chambers, a known number of neutrophils were aliquoted into eight wells of the lower chamber to be used for creating the standard curve (linear range of 10^3^–4 × 10^4^ neutrophils in 30 µL of chemotaxis media). Thirty microliters of the chemotaxis media containing the indicated test samples or control 1% DMSO and 1 nM *f*MLF as the chemoattractant was aliquoted into the remaining wells of the lower chamber, except for 3 lower wells that were reserved for background controls (DMSO treated cells in the upper wells and DMSO without *f*MLF in the lower wells). The lower chamber plate was then covered with the upper filter plate. The pretreated cells were then transferred from the 96-well Greiner plate into the upper wells of the chemotaxis chamber (4 × 10^4^ cells/well in 20 µL), except for the 3 background control wells indicated above and the 8 upper wells corresponding to the lower wells containing neutrophils used for the standard curve. The cells were then allowed to migrate from the upper wells through the polycarbonate membrane filter for 60 min at 37 °C/5% CO_2_. Any remaining unmigrated neutrophils were wiped from the upper membrane in each well using filter paper, and 20 µL of 2.5 mM EDTA was added to each well to detach any cells that migrated through the filter membrane but were still attached to the lower membrane surface (10 min incubation at 4 °C). The number of migrated cells was then determined by measuring the ATP in lysates of transmigrated cells and comparing this to the standard curve obtained using known neutrophil numbers, as described above. Calculation of median effective concentrations (IC_50_) was performed by nonlinear regression analysis of the dose-response curves.

### 3.10. Cytotoxicity Assays

We analyzed monocyte cytotoxicity using THP-1 monocytic cells using a CellTiter-Glo Luminescent Cell Viability Assay Kit (Promega, Madison, WI, USA). Briefly, the cells were incubated (10^4^ cells/well) with the indicated concentrations of the essential oil or compound for 90 min or 24 h at 37 °C/5% CO_2_. After incubation, we added the substrate. The samples were analyzed using a Fluoroscan Ascent FL microplate reader.

### 3.11. Antimicrobial Activity

The standard broth dilution method was used to determine the minimum inhibitory concentration (MIC). The following strains were selected for the testing and were obtained through BEI Resources, NIAID, NIH: *Escherichia coli K-12* (Strain DC10B, NR-49804), *Shigella sonnei* (Strain WRAIR I Virulent, NR-519), *Shigella flexneri* (Strain 24570, NR-517), *Salmonella enterica* subsp. *enterica* (Strain MDCH01 (Serovar Tennessee), NR-20742), *Pseudomonas aeruginosa* (Strain Shr42, NR-48982), *Staphylococcus epidermidis* (Strain M0881, NR-41888), *Staphylococcus aureus* (Strain Sa1912, NR-51347), *Streptococcus pyogenes* (Strain MGAS9882, NR-15272), *Streptococcus pneumoniae* (Strain NP112, NR-19213), and *Bacillus cereus* (Strain Tor 16585, NR-12151) and through BEI Resources, NIAID, NIH as part of the Human Microbiome Project: *Klebsiella pneumoniae* subsp. *Pneumoniae* (Strain WGLW3, HM-748), *Citrobacter freundii* (Strain GED7749C, HM-1280), and *Enterococcus faecalis* (Strain TX2137, HM-432). The assay was performed with a 1% EO stock solution in DMSO (~2500 µg/mL). The EO stock solution was serially diluted in a 96-well plate with TSB broth that had been inoculated with bacterial species. Plates were sealed with sterile gas exchange film and incubated at 37 °C overnight. Optical density was measured at 600 nm using a Thermo Scientific Spectronic 200 spectrophotometer. Assays were performed in quadruplicate.

### 3.12. Molecular Modeling

The PharmMapper Server was utilized for exploring the potential protein targets for (+)-δ-cadinene [77]. The 3D structure of (+)-δ-cadinene was obtained from the PubChem database (https://pubchem.ncbi.nlm.nih.gov/441005; accessed on 7 September 2022). The pharmacophore mapping was performed with the “Human Protein Targets Only” database containing 2241 targets. The top 300 potential human protein targets were retrieved and sorted by the normalized fit score.

### 3.13. Statistical Analysis

Statistical analysis was performed using GraphPad Prism 9 for Windows (GraphPad Software, San Diego, CA, USA). A *p* value of less than 0.05 was considered statistically significant.

## 4. Conclusions

The flower EOs of *E*. *purpurea* mainly comprised germacrene D (42.0 ± 4.61%), α-phellandrene (10.09 ± 1.59%), β-caryophyllene (5.75 ± 1.72%), γ-curcumene (5.03 ± 1.96%), α-pinene (4.44 ± 1.78%), δ-cadinene (3.31 ± 0.61%), and β-pinene (2.43 ± 0.98%). Analysis of the *E. purpurea* EO microbicidal activity showed relatively high activity against a variety of bacterial pathogens. Further analysis showed that the *E. purpurea* EO, germacrene D, α-phellandrene, β-caryophyllene, and δ-cadinene all induced intracellular Ca^2+^ mobilization in human neutrophils, suggesting that they also exhibited innate immunomodulatory activity, as intracellular Ca^2+^ mobilization is a key component of neutrophil activation. Indeed, pretreatment of cells with the *E. purpurea* EO or δ-cadinene inhibited subsequent heterologous agonist-induced Ca^2+^ mobilization and inhibited human neutrophil chemotaxis toward *N*-formyl peptide. This study shows that the *E. purpurea* EO and major components were microbicidal but also had immunomodulatory effects on neutrophil activation, and we suggest that this combination of host defense against pathogens and modulation of the inflammatory response may contribute to the reported beneficial health effects of *Echinacea* extracts. Furthermore, we identified δ-cadinene as one of the active components in *E. purpurea* oil, and pharmacophore mapping studies predicted two potential MAPK targets for (+)-δ-cadinene.

## Figures and Tables

**Table 1:** Chemical composition of five samples (E1–5) of the flower essential oil of *Echinacea purpurea*, expressed as percent (%).

RI_exp_ ^a^	Compound	E1	E2	E3	E4	E5	Average	SD
881	2-Butyl furan	-	-	-	0.06	-	0.06	-
924	α-Thujene	-	-	-	0.05	-	0.05	-
932	α-Pinene	1.86	2.71	1.52	2.58	1.77	2.09	0.53
949	Camphene	-	-	-	0.05	-	0.05	-
971	Sabinene	0.28	0.54	0.26	0.48	0.28	0.37	0.13
977	β-Pinene	1.70	2.82	1.64	2.65	1.64	2.09	0.59
988	Myrcene	0.62	0.86	0.76	0.73	0.57	0.71	0.12
1007	α-Phellandrene	8.95	11.86	8.66	12.09	8.62	10.04	1.78
1024	*p*-Cymene	1.62	3.17	1.57	3.01	1.65	2.20	0.81
1028	Limonene	0.40	0.63	0.44	0.56	0.39	0.48	0.11
1030	β-Phellandrene	0.08	0.13	0.09	0.11	0.07	0.10	0.02
1330	Bicycloelemene	-	-	0.10	-	0.08	0.09	0.01
1335	δ-Elemene	-	-	0.08	0.07	-	0.08	0.01
1345	α-Cubebene	-	-	0.10	0.05	0.10	0.08	0.03
1367	α-Ylangene	-	-	0.14	-	0.14	0.14	-
1374	α-Copaene	0.88	0.89	0.81	0.77	0.93	0.86	0.06
1386	β-Cubebene	0.45	0.46	0.44	-	0.47	0.46	0.01
1388	β-Elemene	1.55	1.77	1.60	2.07	1.56	1.71	0.22
1405	β-Maaliene	-	-	-	-	0.07	0.07	-
1409	α-Gurjunene	-	-	-	0.08	-	0.08	-
1419	β-Caryophyllene	6.31	6.57	6.23	6.60	6.52	6.45	0.17
1428	β-Copaene	0.78	0.61	0.66	-	0.66	0.68	0.07
1429	γ-Elemene	-	-	-	0.16	-	0.16	-
1431	trans-α-Bergamotene	0.62	0.61	0.55	1.02	0.62	0.68	0.19
1447	Z-Muurola-3,5-diene	-	-	0.09	0.09	0.12	0.10	0.02
1447	Isogermacrene D	-	-	-	0.11	-	0.11	-
1449	E-Muurola-3,5-diene	0.14	-	0.14	0.12	0.12	0.13	0.01
1454	α-Humulene	1.90	1.91	1.85	1.86	1.83	1.87	0.03
1458	*allo*-Aromadendrene	0.15	0.20	0.21	0.28	0.17	0.20	0.05
1460	Z-Muurola-4(14),5-diene	0.29	-	0.14	-	0.27	0.23	0.08
1465	Z-Cadina-1(6),4-diene	-	0.23	-	0.19	-	0.21	0.03
1472	Citronellol isobutanoate	1.03	1.23	1.44	1.09	-	1.20	0.18
1474	Dodecenol	0.72	-	-	-	0.92	0.82	0.14
1476	E-Cadina-1(6),4-diene	0.40	0.20	0.49	0.81	0.32	0.44	0.23
1479	γ-Curcumene	3.42	7.44	6.44	2.79	5.05	5.03	1.96
1484	Germacrene D	45.07	33.13	43.84	41.35	45.65	41.81	5.12
1488	β-Selinene	0.27	0.21	0.26	0.44	0.25	0.29	0.09
1492	1-Pentadecene	-	0.81	-	0.55	1.09	0.82	0.27
1494	Bicyclogermacrene	1.73	1.55	1.53	1.93	1.20	1.59	0.27
1496	E-Muurola-4(14),5-diene	0.54	0.41	1.16	0.23	-	0.59	0.40
1497	α-Muurolene	1.19	0.91	1.33	-	1.23	1.17	0.18
1501	β-Cadinene	0.18	0.13	0.20	0.24	0.17	0.18	0.04
1506	E,E-α-Farnesene	-	-	0.09	-	-	0.09	-
1509	Shyobunone	0.17	0.18	0.88	0.10	0.07	0.28	0.34
1510	Cubebol	0.16	0.19	-	-	-	0.18	0.02
1512	γ-Cadinene	0.94	0.73	0.89	0.77	0.85	0.84	0.09
1517	δ-Cadinene	3.89	3.03	4.14	2.99	3.34	3.48	0.52
1524	Isoshyobunone	-	-	-	0.39	0.21	0.30	0.13
1524	Zonarene	-	-	-	0.29	-	0.29	-
1531	E-Cadina-1,4-diene	0.20	0.16	0.21	-	0.19	0.19	0.02
1535	α-Cadinene	0.27	0.21	0.23	0.23	0.23	0.23	0.02
1543	α-Elemol	0.12	0.16	-	-	-	0.14	0.03
1556	α-Cadinol	2.08	1.77	1.62	1.87	1.96	1.86	0.18
1557	Germacrene B	0.33	0.33	0.34	0.39	0.32	0.34	0.03
1560	E-Nerolidol	0.66	0.56	0.58	0.41	0.58	0.56	0.09
1566	1,5-Epoxy salvial-4(14)-ene	0.42	0.39	0.28	0.41	0.39	0.38	0.06
1576	Germacrene D-4-ol	1.14	1.33	0.80	1.06	1.00	1.07	0.19
1580	Caryophyllene oxide	0.82	1.14	0.60	1.63	0.75	0.99	0.41
1583	β-Copaen-4-α-ol	0.23	0.23	0.19	-	0.23	0.22	0.02
1591	Salvial-4(14)-en-1-one	0.27	0.23	0.19	0.28	0.28	0.25	0.04
1608	Humulene epoxide II	0.30	0.35	0.11	0.29	0.18	0.25	0.10
1616	Junenol	0.38	0.32	-	-	-	0.35	0.04
1621	Widdrol isomer	0.25	0.31	0.29	-	0.39	0.31	0.06
1626	1-epi-Cubenol	0.20	0.17	0.14	-	0.17	0.17	0.02
1641	δ-Cadinol	0.33	0.24	0.16	-	-	0.24	0.09
1642	τ-Cadinol	-	-	-	-	0.60	0.60	-
1644	τ-Muurolol	-	-	-	-	0.96	0.96	-
1646	epi-α-Cadinol	0.73	0.57	0.58	0.60	-	0.62	0.07
1649	epi-α-Muurolol	0.83	0.62	0.76	0.75	-	0.74	0.09
1653	Eudesm-4(15),7-dien-1 a-ol	0.08	0.20	-	-	-	0.14	0.08
1657	7E-Tetradecenol	-	0.16	-	-	-	0.16	-
1679	epi-α-Bisabolol	-	0.13	-	-	-	0.13	-
1679	Germacra-4(15),5,10(14)-trien-1-α-ol	0.22	0.13	-	-	-	0.18	0.06
1685	Germacra-4(15),5,10(14)-trien-1-β-ol	0.21	0.18	-	-	-	0.20	0.02
1688	Shyobunol	1.26	1.64	-	-	-	1.45	0.27
1773	14-oxy-α-Muurolene	-	-	-	0.10	-	0.10	-
1828	Z-Thujopsenic acid	0.26	0.26	0.14	0.37	0.27	0.26	0.08
1839	Phytone	0.12	0.15	0.08	0.13	0.15	0.13	0.03
2293	Tricosane	-	-	-	0.03	-	0.03	-
2493	Pentacosane	-	-	-	0.04	-	0.04	-
2692	Heptacosane	-	-	-	0.05	-	0.05	-
2891	Nonacosane	-	-	-	0.03	-	0.03	-
	Unidentified	0.00	1.94	1.93	1.52	2.35		

^a^ Experimental retention index determined with respect to a homologous series of *n*-alkanes on a ZB-5ms column.

**Table 2:** Enantiomeric distributions of chiral compounds present in essential oils of *Echinacea purpurea*.

Chiral Compounds	Average (%)		SD
	(+)	(−)	
α-Pinene	4.11	95.89	2.71
Sabinene	20.43	79.57	4.15
β-Pinene	1.23	98.77	0.41
α-Phellandrene	100.00	0.00	0.00
Limonene	49.21	50.79	10.80
α-Copaene	100.00	0.00	0.00
β-Phellandrene	48.37	51.63	0.10
β-Elemene	23.30	76.70	1.24
β-Caryophyllene	0.00	100.00	0.00
Germacrene D	9.50	90.50	2.45
δ-Cadinene	98.60	1.40	0.82

**Table 3:** Summary of antimicrobial data of *E. purpurea* flower EO.

Bacterial Species	MIC (μg/mL)
*Citrobacter freundii*	312.5 ± 0.49
*Escherichia coli*	625.0 ± 0.32
*Klebsiella pneumoniae*	312.5 ± 0.34
*Bacillus cereus*	312.5 ± 0.43
*Pseudomonas aeruginosa*	312.5 ± 0.44
*Shigella flexneri*	312.5 ± 0.14
*Staphylococcus aureus*	1250.0 ± 0.28
*Stroptococcus pyogenes*	625.0 ± 0.21
*Alcaligenes faecalis*	312.5 ± 0.38
*Staphylococcus epidermidis*	625.0 ± 0.29
*Shigella sonnei*	312.5 ± 0.24
*Enterococcus faecalis*	312.5 ± 0.30

Values are shown as the average MIC ± SD of 3 measurements.

**Table 4:** Effect of *E. purpurea* EO and pure major components on [Ca^2+^]_i_ and chemotaxis in human neutrophils and cytotoxicity in THP-1 monocytic cells.

Essential Oil or Pure Compound	Activation of [Ca^2+^]_i_	Inhibition of *f*MLF-Induced [Ca^2+^]_i_	Chemotaxis	Cytotoxicity (at 24 h)	Cytotoxicity (at 90 min)
	**EC_50_ (μg/mL)**	**IC_50_ (μg/mL)**	**IC_50_ (μg/mL)**		**IC_50_ (μg/mL)**
Echinacea EO	19.9 ± 4.5	1.8 ± 0.6	1.7 ± 0.7	25–30% (at 50 μg/mL)	n.t.
	**EC_50_ (μM)**	**IC_50_ (μM)**	**IC_50_ (μM)**		**IC_50_ (μM)**
β-Caryophyllene	N.A.	0.13 ± 0.02	17.6 ± 5.7 *	n.t.	n.t.
(+)-δ-Cadinene	24.6 ± 6.7	0.48 ± 0.1	7.2 ± 1.6	n.t.	n.t.
α-Phellandrene	20.8 ± 7.5	7.9 ± 1.1	19.4 ± 1.5	35% at 50 μM	n.t.
Germacrene D	21.7 ± 7.1	1.9 ± 0.1	5.4 ± 2.3 *	n.t.	n.t.

EC_50_ and IC_50_ values were determined by nonlinear regression analysis of the dose-response curves, as described under Section 3. * Values are as reported previously [70]. For cytotoxicity assays, THP-1 cells were incubated with indicated concentrations of the compounds for 90 min and 24 h and cell viability was analyzed. N.A. and n.t. indicate the samples had essentially no activity or no cytotoxicity, respectively (EC_50_ or IC_50_ > 55 µM for pure compounds or >55 µg/mL for the essential oils). The data are presented as the mean ± SD of three independent experiments.

**Table 5:** Potential human protein targets of (+)-δ-cadinene identified by PharmMapper.

Rank	PDB ID	Target Name	Fit Score
**1**	1J96	Aldo-keto reductase family 1 member C2	2.974
**2**	1REU	Bone morphogenetic protein 2	2.948
**3**	1PME	Mitogen-activated protein kinase 1	2.918
**4**	1P49	Steryl-sulfatase	2.905
**5**	1F40	Peptidyl-prolyl cis-trans isomerase FKBP1A	2.901
**6**	1E7E	Serum albumin	2.874
**7**	1SHJ	Caspase-7	2.842
**8**	2PIN	Thyroid hormone receptor beta	2.837
**9**	2P3G	MAP kinase-activated protein kinase 2	2.804
**10**	1L6L	Apolipoprotein A-II	2.763

## Data Availability

Data is contained within the article.

## References

[1] Shemluck M. (1982). Medicinal and Other Uses of the Compositae by Indians in the United States and Canada. J. Ethnopharmacol..

[2] Mohamed Sharif K.O., Tufekci E.F., Ustaoglu B., Altunoglu Y.C., Zengin G., Llorent-Martínez E.J., Guney K., Baloglu M.C. (2021). Anticancer and Biological Properties of Leaf and Flower Extracts of *Echinacea purpurea* (L.) Moench. Food Biosci..

[3] WFO Plant List: *Echinacea purpurea* (L.) Moench. https://wfoplantlist.org/plant-list/taxon/wfo-0000036347-2022-12?page=1.

[4] Moltó J., Valle M., Miranda C., Cedeño S., Negredo E., Clotet B. (2012). Herb-Drug Interaction between *Echinacea purpurea* and Etravirine in HIV-Infected Patients. Antimicrob. Agents Chemother..

[5] Ogal M., Johnston S.L., Klein P., Schoop R. (2021). Echinacea Reduces Antibiotic Usage in Children through Respiratory Tract Infection Prevention: A Randomized, Blinded, Controlled Clinical Trial. Eur. J. Med. Res..

[6] Linde K., Barrett B., Wölkart K., Bauer R., Melchart D. (2006). Echinacea for Preventing and Treating the Common Cold. Cochrane Database Syst. Rev..

[7] Temerdashev Z., Vinitskaya E., Meshcheryakova E., Shpigun O. (2022). Chromatographic Analysis of Water and Water-Alcohol Extracts of *Echinacea purpurea* L. Obtained by Various Methods. Microchem. J..

[8] Hu C., Kitts D. (2000). Studies on the Antioxidant Activity of Echinacea Root Extract. J. Agric. Food Chem..

[9] Haller J., Krecsak L., Zámbori J. (2020). Double-Blind Placebo Controlled Trial of the Anxiolytic Effects of a Standardized Echinacea Extract. Phytother. Res..

[10] Bauer R. (2002). New Findings on the Pharmacological Activity and Therapeutical Efficacy of Preparations of the Pressed Juice of *Echinacea purpurea*. Wien. Med. Wochenschr..

[11] Ardjomand-Woelkart K., Bauer R. (2015). Review and Assessment of Medicinal Safety Data of Orally Used Echinacea Preparations. Planta Med..

[12] Yu D., Yuan Y., Jiang L., Tai Y., Yang X., Hu F., Xie Z. (2013). Anti-Inflammatory Effects of Essential Oil in *Echinacea purpurea* L.. Pak. J. Pharm. Sci..

[13] Xu W., Hu B., Cheng Y., Guo Y., Yao W., Qian H. (2022). *Echinacea purpurea* Suppresses the Cell Survival and Metastasis of Hepatocellular Carcinoma through Regulating the PI3K/Akt Pathway. Int. J. Biochem. Cell Biol..

[14] Miller S.C. (2005). *Echinacea*: A Miracle Herb against Aging and Cancer? Evidence in vivo in Mice. Evid.-Based Complement. Altern. Med..

[15] Barrett B., Brown R., Rakel D., Mundt M., Bone K., Barlow S., Ewers T. (2010). Echinacea for Treating the Common Cold: A Randomized Controlled Trial. Ann. Intern. Med..

[16] Karsch-Völk M., Kiefer B.B., Bauer R., Linde A.-W.K. (2014). Echinacea for Preventing and Treating the Common Cold (Review). Cochrane Database Syst. Rev..

[17] Nahas R., Balla A. (2011). Clinical Review Complementary and Alternative Medicine for Prevention and Treatment of the Common Cold. Can. Fam. Physician.

[18] Jawad M., Schoop R., Suter A., Klein P., Eccles R. (2012). Safety and Efficacy Profile of *Echinacea purpurea* to Prevent Common Cold Episodes: A Randomized, Double-Blind, Placebo-Controlled Trial. Evid.-Based Complement. Altern. Med..

[19] Ross S.M. (2016). *Echinacea purpurea*: A Proprietary Extract of *Echinacea purpurea* Is Shown to Be Safe and Effective in the Prevention of the Common Cold. Holist. Nurs. Pract..

[20] Schapowal A., Klein P., Johnston S.L. (2015). Echinacea Reduces the Risk of Recurrent Respiratory Tract Infections and Complications: A Meta-Analysis of Randomized Controlled Trials. Adv. Ther..

[21] Vimalanathan S., Schoop R., Suter A., Hudson J. (2017). Prevention of Influenza Virus Induced Bacterial Superinfection by Standardized *Echinacea purpurea*, via Regulation of Surface Receptor Expression in Human Bronchial Epithelial Cells. Virus Res..

[22] Isbaniah F., Wiyono W.H., Yunus F., Setiawati A., Totzke U., Verbruggen M.A. (2011). *Echinacea purpurea* along with Zinc, Selenium and Vitamin C to Alleviate Exacerbations of Chronic Obstructive Pulmonary Disease: Results from a Randomized Controlled Trial. J. Clin. Pharm. Ther..

[23] Weber W., Taylor J.A., Vander Stoep A., Weiss N.S., Standish L.J., Calabrese C. (2005). *Echinacea purpurea* for Prevention of Upper Respiratory Tract Infections in Children. J. Altern. Complement. Med..

[24] Signer J., Jonsdottir H.R., Albrich W.C., Strasser M., Züst R., Ryter S., Ackermann-Gäumann R., Lenz N., Siegrist D., Suter A. (2020). In Vitro Virucidal Activity of Echinaforce®, an *Echinacea purpurea* Preparation, against Coronaviruses, Including Common Cold Coronavirus 229E and SARS-CoV-2. Virol. J..

[25] Pleschka S., Stein M., Schoop R., Hudson J.B. (2009). Anti-Viral Properties and Mode of Action of Standardized *Echinacea purpurea* Extract against Highly Pathogenic Avian Influenza Virus (H5N1, H7N7) and Swine-Origin H1N1 (S-OIV). Virol. J..

[26] Ladenheim D., Horn O., Werneke U., Phillpot M., Murungi A., Theobald N., Orkin C. (2008). Potential Health Risks of Complementary Alternative Medicines in HIV Patients. HIV Med..

[27] Kolev E., Mircheva L., Edwards M.R., Johnston S.L., Kalinov K., Stange R., Gancitano G., Berghe W.V., Kreft S. (2022). *Echinacea purpurea* For the Long-Term Prevention of Viral Respiratory Tract Infections During Covid-19 Pandemic: A Randomized, Open, Controlled, Exploratory Clinical Study. Front. Pharmacol..

[28] Lee T.-T., Huang C.-C., Shieh X.-H., Chen C.-L., Chen L.-J., Yu B. (2010). Flavonoid, Phenol and Polysaccharide Contents of *Echinacea purpurea* L. and Its Immunostimulant Capacity In Vitro. Int. J. Environ. Sci. Dev..

[29] Sharma M., Schoop R., Suter A., Hudson J.B. (2011). The Potential Use of Echinacea in Acne: Control of Propionibacterium Acnes Growth and Inflammation. Phytother. Res..

[30] Oláh A., Szabó-Papp J., Soeberdt M., Knie U., Dähnhardt-Pfeiffer S., Abels C., Bíró T. (2017). *Echinacea purpurea*-Derived Alkylamides Exhibit Potent Anti-Inflammatory Effects and Alleviate Clinical Symptoms of Atopic Eczema. J. Dermatol. Sci..

[31] Dogan Z., Ergul B., Sarikaya M., Filik L., Gonultaş A. (2014). The Protective Effect of *Echinacea* spp. (*Echinacea angustifolia* and *Echinacea purpurea*) in a Rat Colitis Model Induced by Acetic Acid. Pak. J. Pharm. Sci..

[32] Bauer R., Lawson L., Bauer R. (1998). *Echinacea*: Biological Effects and Active Principles. Phytomedicines of Europe: Chemistry and Biological Activity.

[33] Rininger J.A., Kickner S., Chigurupati P., McLean A., Franck Z. (2000). Immunopharmacological Activity of Echinacea Preparations Following Simulated Digestion on Murine Macrophages and Human Peripheral Blood Mononuclear Cells. J. Leukoc. Biol..

[34] Nyalambisa M., Oyemitan I.A., Matewu R., Oyedeji O.O., Oluwafemi O.S., Songca S.P., Nkeh-Chungag B.N., Oyedeji A.O. (2017). Volatile Constituents and Biological Activities of the Leaf and Root of Echinacea Species from South Africa. Saudi Pharm. J..

[35] Oüzek G., Schepetkin I.A., Utegenova G.A., Kirpotina L.N., Andrei S.R., Oüzek T., Baser K.H.C., Abidkulova K.T., Kushnarenko S.V., Khlebnikov A.I. (2017). Chemical Composition and Phagocyte Immunomodulatory Activity of *Ferula iliensis* Essential Oils. J. Leukoc. Biol..

[36] Chouhan S., Sharma K., Guleria S. (2017). Antimicrobial Activity of Some Essential Oils—Present Status and Future Perspectives. Medicines.

[37] Harborne J., Williams C., Miller S.C., Yu H. (2004). Phytochemistry of the Genus Echinacea. Echinacea: The Genus Echinacea (Medicinal and Aromatic Plants—Industrial Profiles).

[38] Barnes J., Anderson L.A., Gibbons S., Phillipson J.D. (2010). *Echinacea* Species (*Echinacea angustifolia* (DC.) Hell., *Echinacea pallida* (Nutt.) Nutt., *Echinacea purpurea* (L.) Moench): A Review of Their Chemistry, Pharmacology and Clinical Properties. J. Pharm. Pharmacol..

[39] Lin Z., Neamati N., Zhao H., Kiryu Y., Turpin J.A., Aberham C., Strebel K., Kohn K., Witvrouw M., Pannecouque C. (1999). Chicoric Acid Analogues as HIV-1 Integrase Inhibitors. J. Med. Chem..

[40] Lee J., Scagel C.F. (2013). Chicoric Acid: Chemistry, Distribution, and Production. Front. Chem..

[41] Parsons J.L., Liu R., Smith M.L., Harris C.S. (2018). Echinacea Fruit: Phytochemical Localization and Germination in Four Species of *Echinacea*. Botany.

[42] Sharifi-Rad M., Mnayer D., Morais-Braga M.F.B., Carneiro J.N.P., Bezerra C.F., Coutinho H.D.M., Salehi B., Martorell M., del Mar Contreras M., Soltani-Nejad A. (2018). *Echinacea* Plants as Antioxidant and Antibacterial Agents: From Traditional Medicine to Biotechnological Applications. Phytother. Res..

[43] Cozzolino R., Malvagna P., Spina E., Giori A., Fuzzati N., Anelli A., Garozzo D., Impallomeni G. (2006). Structural Analysis of the Polysaccharides from Echinacea Angustifolia Radix. Carbohydr. Polym..

[44] Pellati F., Epifano F., Contaldo N., Orlandini G., Cavicchi L., Genovese S., Bertelli D., Benvenuti S., Curini M., Bertaccini A. (2011). Chromatographic Methods for Metabolite Profiling of Virus- and Phytoplasma-Infected Plants of *Echinacea purpurea*. J. Agric. Food Chem..

[45] Hudaib M., Bellardi M.G., Rubies-Autonell C., Fiori J., Cavrini V. (2001). Chromatographic (GC-MS, HPLC) and Virological Evaluations of Salvia Sclarea Infected by BBWV-I. Farmaco.

[46] Kaya M., Merdivan M., Tashakkori P., Erdem P., Anderson J.L. (2019). Analysis of Echinacea Flower Volatile Constituents by HS-SPME-GC/MS Using Laboratory-Prepared and Commercial SPME Fibers. J. Essent. Oil Res..

[47] Mirjalili M.H., Salehi P., Badi H.N., Sonboli A. (2006). Volatile Constituents of the Flowerheads of ThreeEchinacea Species Cultivated in Iran. Flavour Fragr. J..

[48] Mazza G., Cottrell T. (1999). Volatile Components of Roots, Stems, Leaves, and Flowers of *Echinacea* Species. J. Agric. Food Chem..

[49] Vaverková S., Mikulásová M., Habán M., Tekel’ J., Hollá M., Otepka P. (2007). Variability of the Essential Oil from Three Sorts of Echinacea MOENCH Genus during Ontogenesis. Ceska Slov. Farm..

[50] Yazdanian M., Rostamzadeh P., Alam M., Abbasi K., Tahmasebi E., Tebyaniyan H., Ranjbar R., Seifalian A., Moghaddam M.M., Kahnamoei M.B. (2022). Evaluation of Antimicrobial and Cytotoxic Effects of Echinacea and Arctium Extracts and Zataria Essential Oil. AMB Express.

[51] Pérez Zamora C., Torres C., Nuñez M. (2018). Antimicrobial Activity and Chemical Composition of Essential Oils from Verbenaceae Species Growing in South America. Molecules.

[52] Leite-Sampaio N.F., Gondim C.N.F.L., Martins R.A.A., Siyadatpanah A., Norouzi R., Kim B., Sobral-Souza C.E., Gondim G.E.C., Ribeiro-Filho J., Coutinho H.D.M. (2022). Potentiation of the Activity of Antibiotics against ATCC and MDR Bacterial Strains with (+)-α-Pinene and (-)-Borneol. BioMed Res. Int..

[53] Yoo H.-J., Jwa S.-K. (2018). Inhibitory Effects of β-Caryophyllene on Streptococcus Mutans Biofilm. Arch. Oral Biol..

[54] Moo C.-L., Yang S.-K., Osman M.-A., Yuswan M.H., Loh J.-Y., Lim W.-M., Lim S.-H.-E., Lai K.-S. (2020). Antibacterial Activity and Mode of Action of β-Caryophyllene on *Bacillus cereus*. Pol. J. Microbiol..

[55] Dahham S., Tabana Y., Iqbal M., Ahamed M., Ezzat M., Majid A., Majid A. (2015). The Anticancer, Antioxidant and Antimicrobial Properties of the Sesquiterpene β-Caryophyllene from the Essential Oil of Aquilaria Crassna. Molecules.

[56] da Silva A.C.R., Lopes P.M., de Azevedo M.M.B., Costa D.C.M., Alviano C.S., Alviano D.S. (2012). Biological Activities of A-Pinene and β-Pinene Enantiomers. Molecules.

[57] de Souza W.F.C., de Lucena F.A., de Castro R.J.S., de Oliveira C.P., Quirino M.R., Martins L.P. (2021). Exploiting the Chemical Composition of Essential Oils from Psidium Cattleianum and Psidium Guajava and Its Antimicrobial and Antioxidant Properties. J. Food Sci..

[58] Radice M., Durofil A., Buzzi R., Baldini E., Martínez A.P., Scalvenzi L., Manfredini S. (2022). Alpha-Phellandrene and Alpha-Phellandrene-Rich Essential Oils: A Systematic Review of Biological Activities, Pharmaceutical and Food Applications. Life.

[59] Adolpho L.O., Paz L.H.A., Rosa O., Morel A.F., Dalcol I.I. (2023). Chemical Profile and Antimicrobial Activity of *Leonotis nepetifolia* (L.) R. Br. Essential Oils. Nat. Prod. Res..

[60] Cárdenas J., Rojas J., Rojas-Fermin L., Lucena M., Buitrago A. (2012). Essential Oil Composition and Antibacterial Activity of *Monticalia greenmaniana* (Asteraceae). Nat. Prod. Commun..

[61] Uçüncü O., Kahriman N., Terzioğlu S., Karaoğlue S.A., Yayli N. (2010). Composition and Antimicrobial Activity of the Essential Oils from Flowers of *Senecio othonnae*, *S. racemosus*, and *S. nemorensis*. Nat. Prod. Commun..

[62] González A.M., Tracanna M.I., Amani S.M., Schuff C., Poch M.J., Bach H., Catalán C.A.N. (2012). Chemical Composition, Antimicrobial and Antioxidant Properties of the Volatile Oil and Methanol Extract of *Xenophyllum poposum*. Nat. Prod. Commun..

[63] Hoi T.M., Chung N.T., Huong L.T., Ogunwande I.A. (2021). Studies on Asteraceae: Chemical Compositions of Essential Oils and Antimicrobial Activity of the Leaves of *Vernonia patula* (Dryand.) Merr. and *Grangea maderaspatana* (L.) Poir. from Vietnam. J. Essent. Oil Bear. Plants.

[64] Thinh B.B., Thin D.B. (2023). Essential Oil Composition, Antimicrobial and Antioxidant Properties of *Pluchea eupatorioides* Kurz Collected from Vietnam. J. Essent. Oil Bear. Plants.

[65] Kim H.-R., Oh S.-K., Lim W., Lee H.K., Moon B.-I., Seoh J.-Y., Commun N.P. (2014). Immune Enhancing Effects of *Echinacea purpurea* Root Extract by Reducing Regulatory T Cell Number and Function. Nat. Prod. Commun..

[66] Park S.J., Lee M., Kim D., Oh D.H., Prasad K.S., Eun S., Lee J. (2021). *Echinacea purpurea* Extract Enhances Natural Killer Cell Activity in Vivo by Upregulating MHC II and Th1-Type CD4+T Cell Responses. J. Med. Food.

[67] Fonseca F.N., Papanicolaou G., Lin H., Lau C.B.S., Kennelly E.J., Cassileth B.R., Cunningham-Rundles S. (2014). *Echinacea purpurea* (L.) Moench Modulates Human T-Cell Cytokine Response. Int. Immunopharmacol..

[68] Malech H.L., DeLeo F.R., Quinn M.T. (2014). The Role of Neutrophils in the Immune System: An Overview. Neutrophil Methods Protoc..

[69] Dixit N., Kim M.-H., Rossaint J., Yamayoshi I., Zarbock A., Simon S.I. (2012). Leukocyte Function Antigen-1, Kindlin-3, and Calcium Flux Orchestrate Neutrophil Recruitment during Inflammation. J. Immunol..

[70] Gronski M.A., Kinchen J.M., Juncadella I.J., Franc N.C., Ravichandran K.S. (2009). An Essential Role for Calcium Flux in Phagocytes for Apoptotic Cell Engulfment and the Anti-Inflammatory Response. Cell Death Differ..

[71] Schepetkin I., Özek G., Özek T., Kirpotina L., Khlebnikov A., Quinn M. (2020). Chemical Composition and Immunomodulatory Activity of Hypericum Perforatum Essential Oils. Biomolecules.

[72] Schepetkin I.A., Kushnarenko S.V., Özek G., Kirpotina L.N., Sinharoy P., Utegenova G.A., Abidkulova K.T., Özek T., Başer K.H.C., Kovrizhina A.R. (2016). Modulation of Human Neutrophil Responses by the Essential Oils from *Ferula akitschkensis* and Their Constituents. J. Agric. Food Chem..

[73] Schepetkin I.A., Kushnarenko S.V., Özek G., Kirpotina L.N., Utegenova G.A., Kotukhov Y.A., Danilova A.N., Özek T., Başer K.H.C., Quinn M.T. (2015). Inhibition of Human Neutrophil Responses by the Essential Oil of *Artemisia kotuchovii* and Its Constituents. J. Agric. Food Chem..

[74] Futosi K., Fodor S., Mócsai A. (2013). Neutrophil Cell Surface Receptors and Their Intracellular Signal Transduction Pathways. Int. Immunopharmacol..

[75] Decarlo A., Johnson S., Ouédraogo A., Dosoky N.S., Setzer W.N. (2019). Chemical Composition of the Oleogum Resin Essential Oils of *Boswellia dalzielii* from Burkina Faso. Plants.

[76] Kumar Poudel D., Dangol S., Rokaya A., Maharjan S., Kumar Ojha P., Rana J., Dahal S., Timsina S., Dosoky N.S., Satyal P. (2022). Quality Assessment of *Zingiber officinale* Roscoe Essential Oil from Nepal. Nat. Prod. Commun..

[77] Liu X., Ouyang S., Yu B., Liu Y., Huang K., Gong J., Zheng S., Li Z., Li H., Jiang H. (2010). PharmMapper Server: A Web Server for Potential Drug Target Identification Using Pharmacophore Mapping Approach. Nucleic Acids Res..

